# Pathogenesis and treatment of chronic rhinosinusitis from the perspective of sinonasal epithelial dysfunction

**DOI:** 10.3389/fmed.2023.1139240

**Published:** 2023-04-17

**Authors:** Yuanqiong He, Yijie Fu, Yuqi Wu, Tianmin Zhu, Hui Li

**Affiliations:** ^1^School of Heath Preservation and Rehabilitation, Chengdu University of Traditional Chinese Medicine, Chengdu, China; ^2^School of Preclinical Medicine, Chengdu University, Chengdu, China

**Keywords:** epithelium, dysfunction, pathogenesis, treatment, chronic rhinosinusitis

## Abstract

**Background:**

Chronic rhinosinusitis (CRS) is a clinical syndrome primarily characterized by long-term mucosal inflammation of the nasal cavity and sinuses. The pathogenesis of CRS is still unclear due to its high heterogeneity. A number of studies have recently focused on the sinonasal epithelium. Thus, there has been a quantum leap in awareness of the role of the sinonasal epithelium, which is now understood as an active functional organ rather than simply an inert mechanical barrier. Undoubtedly, epithelial dysfunction plays a vital role in the onset and development of CRS.

**Objective:**

In this article, we discuss the potential contribution of sinonasal epithelium dysfunction to CRS pathogenesis and explore a few current and developing therapeutic options targeting the sinonasal epithelium.

**Results:**

Impaired mucociliary clearance (MCC) and an abnormal sinonasal epithelial barrier are usually considered to be the main causative factors in CRS. Epithelial-derived bioactive substances, such as cytokines, exosomes, and complements, play a vital role in the regulation of innate and adaptive immunity and contribute to the pathophysiological alterations of CRS. The phenomena of epithelial–mesenchymal transition (EMT), mucosal remodeling, and autophagy observed in CRS offer some novel insights into the pathogenesis of this disease. In addition, existing treatment options targeting disorder of sinonasal epithelium can help to relieve the main symptoms associated with CRS to some extent.

**Conclusion:**

The presence of a normal epithelium is fundamental for maintaining homeostasis in the nasal and paranasal sinuses. Here, we describe various aspects of the sinonasal epithelium and highlight the contributions of epithelial dysfunction to CRS pathogenesis. Our review provides sound evidence of the need for in-depth study of the pathophysiological alterations of this disease and for the development of novel epithelium-targeting alternative treatments.

## Introduction

Chronic rhinosinusitis (CRS), which causes local inflammation of the nasal mucosa and paranasal sinuses, is mainly characterized by symptoms of congestion, stuffiness, facial pressure, and hyposmia, with a duration of at least 3 months. This disease affects ~10% of the global population and can significantly impair their quality of life (1, 2). Based on the presence or absence of nasal polyps (NPs), CRS has been classically divided into two major subgroups, namely, CRS with nasal polyps (CRSwNP) and CRS without nasal polyps (CRSnNP) (3). Moreover, based on the cytokine production patterns of canonical T-cells, recent studies have described three distinct endotypes of patients, as follows: type 1, regulated by Th1 cytokine IFN-γ; type 2, mediated by Th2 cytokines IL-4, IL-5, IL-13, and IL-9; and type 3, controlled by Th17 cytokines IL-17 and IL-22 (4, 5). Thus, given the significant variation found in the presence and extent of eosinophilia in the sinuses, CRS has been classified into two subsets, namely, idiopathic eosinophilic CRS (E-CRS) and non-eosinophilic CRS (NE-CRS). It has been shown that CRS patients with severe tissue eosinophilia usually display severe symptoms and experience poorer treatment outcomes (6).

As CRS is a highly heterogeneous disease, its pathogenesis has not yet been well-elucidated. A number of factors have been found to be associated with the onset and development of CRS, including geographical and racial factors, environmental exposures, sinus microbiota dysbiosis, mucociliary clearance (MCC) dysfunction, epithelial barrier impairment, and disrupted immune response (1, 2, 7, 8). The epithelium is the first line of defense for the nasal mucosa. It has been established that the MCC system and cell–cell contact form the fundamental mechanical barrier. When the epithelial barrier of the sinuses is damaged, its appearance changes substantially, from that of an inert physical barrier to that of an active functional organ (9) that can secrete a wide variety of bioactive substances, such as cytokines and exosomes, as well as complement proteins, and thus facilitates the recruitment and regulation of different immune cells (10–12). Essentially, the sinonasal epithelium plays a key role in both innate and adaptive immunity. However, abnormal capability of the epithelium can have a significant impact on the onset and development of CRS. Epithelial-to-mesenchymal transition (EMT), mucosal remodeling, and autophagy have been commonly observed in CRS, presenting new insight into sinonasal epithelium malfunction; these phenomena might have some connection with the pathogenesis of this disease.

The optimal treatment of CRS has undoubtedly been found to be complicated due to its multifactorial pathogenesis. However, administration of topical or systemic corticosteroids plays a fundamental role in medical treatment of CRS (13). Nasal saline irrigation is an effective adjunct to treatment for relieving the various symptoms of CRS (14). Failure of medical management can often lead patients to undergo surgery. However, this option does not provide effective prevention of relapse of the disease (15). Recently, the effectiveness of biological treatment has been demonstrated, and biologics have emerged as good options for CRS therapy (16). Despite the presence of a wide variety of therapies for this disease, there is still a sizable proportion of patients with CRS who display poor treatment outcomes, which can be mainly attributed to its intricate pathogenesis. In this article, we provide an elegant summary of the contributions of altered epithelial functions to the pathogenesis of CRS and discuss the possible influence of the current treatment regime on the sinonasal epithelium.

## Roles of epithelial dysfunction in the development of CRS

### Barrier dysfunction

The physical-mechanical barrier consists of tight junctions (TJs), adherens junctions, gap junctions, desmosomes, and hemidesmosomes; these components facilitate strong cell–cell contact, form a tight barrier, establish cell polarity, and can effectively regulate the mobility of different ions and molecules (17). TJs consist of zonula occludens-1 (ZO-1), occludin, and claudins, as well as junctional adhesion molecule 1 (JAM-1) proteins (18). Adherens junctions contain the transmembrane proteins E-cadherin and nectin and the intracellular proteins α- and β-catenin (18). Desmosomal attachments consist of the heterotypic binding of desmoglein (DSG1, DSG2) (19).

Interestingly, some studies have observed significantly decreased expression of ZO-1, occludin, claudin-1, JAM-1, DSG1, and DSG2 in the primary epithelial cells of CRS patients as compared with those of healthy controls (20–22). Transepithelial electrical resistance (TER) is one of the indicators reflecting the function of the epithelial barrier (23). Patients with CRSwNP exhibit reduced TER, which could be associated with epithelial barrier damage (21). In patients with E-CRSwNP, E-cadherin expression is markedly downregulated (24). In addition, changes in the sinus microenvironment can also cause barrier damage. CRSwNP is often characterized by type 2 inflammation with high levels of Th2 cytokines, including IL-4 and IL-13 (25). CRSnNP is associated with high levels of Th1 cytokines such as IFN-γ; however, it is noteworthy that IFN-γ has been observed to be highly expressed in NE-CRSwNP (26). Elevation of IL-4, IL-13, and IFN-γ in CRSwNP can cause the downregulation of TJ proteins and reduction of TER, thus leading to epithelial disruption (21, 27, 28).

The interaction of the airway epithelial cells with airborne environmental pollution and bacterial toxins is critical in the impairment of epithelial barrier function. A study on house dust mites (HDMs) has indicated that the compromised epithelial barrier function caused by HDMs is associated with lower expression of both occludin and ZO-1 proteins (29). Moreover, another study has reported that the cigarette smoke extract (CSE) stimulation causes global disruption of the epithelial junctional proteins ZO-1 and JAM-A, along with an associated decrease in TER levels (22). Particulate matter (PM) generated by human activities could be very harmful to the health of the respiratory mucosa. According to *in vitro* studies, exposure of human nasal epithelial cells to PM with a diameter of <2.5 μm can lead to significantly decreased expression of various TJ proteins (claudin-1, occludin, and ZO-1), increased paracellular permeability, and decreased TER, and to an increase in the production of pro-inflammatory cytokines IL-8 and thymic stromal lymphopoietin (TSLP), which can significantly damage the integrity and function of the epithelial barrier (30, 31). Moreover, exposure to PM2.5 can increase levels of intracellular reactive oxygen species (ROS) and the nuclear translocation of NF-E2-related factor-2 (Nrf2), induce oxidative stress and inflammatory responses, and aggravate existing damage to the nasal epithelial barrier (32). In addition, bacterial infections caused by *Staphylococcus aureus* have been detected in airways with compromised barrier function. For instance, one study found that *S. aureus* enterotoxin B (SEB) can activate toll-like receptor 2 (TLR2) and stimulate the subsequent release of the pro-inflammatory cytokines IL-6 and IL-8, which can cause epithelial cells to reduce expression of both occludin and ZO-1 and increase mucosal permeability (33).

When the integrity of the epithelium is destroyed, epithelial permeability can effectively increase. Invading exogenous irritants can thus activate the immune cells and thereby initiate an immune response (34). Selected studies highlighting the destruction of CRS epithelial integrity are summarized in Table 1; selected studies highlighting the various factors affecting epithelial barrier function are summarized in Table 2.

**Table 1:** Studies describing the destruction of CRS epithelial integrity.

**Authors**	**Method**	**Results**
Soyka MB	Nasal mucosal epithelial biopsy of CRSwNP	Reduced TER expression
		Decrease in expression of TJ proteins (occludin, zonula occludens, and claudin-4)
Soyka MB	Stimulation of HNECs with cytokine IL-4	Reduced TER expression
		Reduced TJ protein expression
Wang M	ECRSwNP epithelial tissue	Reduced E-cadherin expression
Li Y	Histopathologic study of sinonasal tissue	Decrease in expression of ZO-1, claudin-1, DSG1, and DSG2

TER, transepithelial electrical resistance; TJ, tight junction; HNECs, human nasal epithelial cells.

**Table 2:** Studies describing the various factors affecting epithelial barrier function.

**Authors**	**Factor**	**Results**
Steelant	HDMs	Decrease in expression of TJ proteins (occludin, zonula occludens −1)
Tharakan A	CES	Decrease in expression of TER and of ZO-1 and JAMA
Xian M, Zhao R, Hong Z	PM	Decrease in expression of TJ proteins (claudin-1, occludin, and ZO-1) and TER; paracellular permeability; increase in ROS
Martens K	SEB	Decrease in expression of TJ proteins (occludin and ZO-1); increase in mucosal permeability

HDMs, house dust mites; CSE, cigarette smoke extract; PM, particulate matter; ROS, reactive oxygen species; SEB, S. aureus enterotoxin B.

### Mucociliary dysfunction

The mucociliary clearance (MCC) system consists of three major functional components, namely, cilia activity, the airway surface liquid (ASL) layer, and mucin secretion. Ciliated epithelial cells mainly function to clear away the various pathogens and inhaled irritants trapped by the airway surface liquid and mucus. A number of prior studies have reported that CRS is related to impairment of MCC, the degree of this impairment being correlated with the severity of CRS (11, 35). Patients with CRS often have a local infection in the nasal and paranasal cavities. In previous studies, bacteria such as *Haemophilus influenzae, Streptococcus pneumoniae, S. aureus, Aspergillus fumagatus*, and *P. aeruginosa* have been demonstrated to release different toxins that can effectively destroy ciliated epithelial cells (36). Interestingly, two distinct parameters, namely, ciliary beating frequency (CBF) and ciliary bend distance (CBD), can potentially be used to evaluate cilia transport capacity. ASL combined with coordinated ciliary beating is a critical component of the mucociliary clearance apparatus. Moreover, studies have found that CBD, CBF, and ASL are regulated by CI^−^. Cl^−^ secretion of the nasal epithelial cells has been found to be significantly reduced in patients with CRS, thus damaging the function of the cilia as well as the ASL and increasing the viscosity of mucus (37–39). Mucin 5AC (MUC5AC), produced by the goblet cells of the airway epithelium, is a major constituent of airway mucus. Epidermal growth factor (EGF) has been found to upregulate MUC5AC expression in mucus secreted by patients with CRS through transmembrane protein 16A (TMEM16A) (40, 41). Similarly, major basic protein (MBP) in the airway mucus can effectively disrupt exogenous pathogens and infected cells. Moreover, researchers have found that the mucus of patients with CRS may exhibit higher expression of MBP in comparison to that of healthy people. Notably, excessive expression of MBP harms the normal host cells and intensifies inflammation of the nasal mucosa (42). Mucociliary dysfunction can weaken protection of the nasal mucosa and has been found to be conducive to bacterial colonization, biofilm formation, and mucositis, which is a non-negligible factor in CRS pathogenesis.

### Roles of epithelial-derived bioactive substances

#### Cytokines

Considering the elevation of canonical T-cell cytokines in this condition, recent studies have described three endotypes of CRS: type 1 is mainly associated with an increase in Th1 cytokines IFN-γ and TNF-α; type 2 is associated with an increase in Th2 cytokines IL-4, IL-5, and IL-13; and type 3 is related to the Th17 cytokines IL-17 and IL-22 (4, 5).

When exposed to various foreign irritants, nasal epithelial cells can secrete a variety of inflammatory cytokines, such as TNF-α, TSLP, IL-25, IL-33, and IL-6 (43). TNF-α is a Th1 cytokine and can promote the release of IL-6, IL-10, and IFN-γ. It is generally believed that CRSsNP mainly involves Th1-type inflammation with high expression of IFN-γ (44). In addition, pro-inflammatory cytokines such as TNF-α and IFN-γ have been reported to induce necroptosis in CRSwNP, which in turn can stimulate the production and release of pro-inflammatory cytokines and the recruitment of neutrophils, thus leading to exacerbation of the inflammation. TNF-α and IFN-γ may act in conjunction, which can cause a vicious cycle of necroptosis and inflammation (45).

Epithelial-derived cytokines TSLP, IL-25, and IL-33 can promote the production of different Th2 cytokines, such as IL-4, IL-5, and IL-13, by activating Th2 cells as well as group 2 innate lymphoid cells (ILC2s), thus further inducing type 2 inflammatory responses (46). A number of studies have shown that the nasal polyp tissues of patients with CRSwNP accumulate more dendritic cells (DCs) expressing IL-17Rb, ST2, and TSLPR than the tissues of control individuals. DCs are activated in response to IL-25, IL-33, and TSLP and can then initiate Th2 reactions (47). Moreover, some recent studies have provided evidence that increased expression of IL-25, IL-33, and TSLP could be correlated with severe tissue eosinophilia, especially in the nasal polyp tissues of patients with CRSwNP (48, 49).

**Figure 1:**
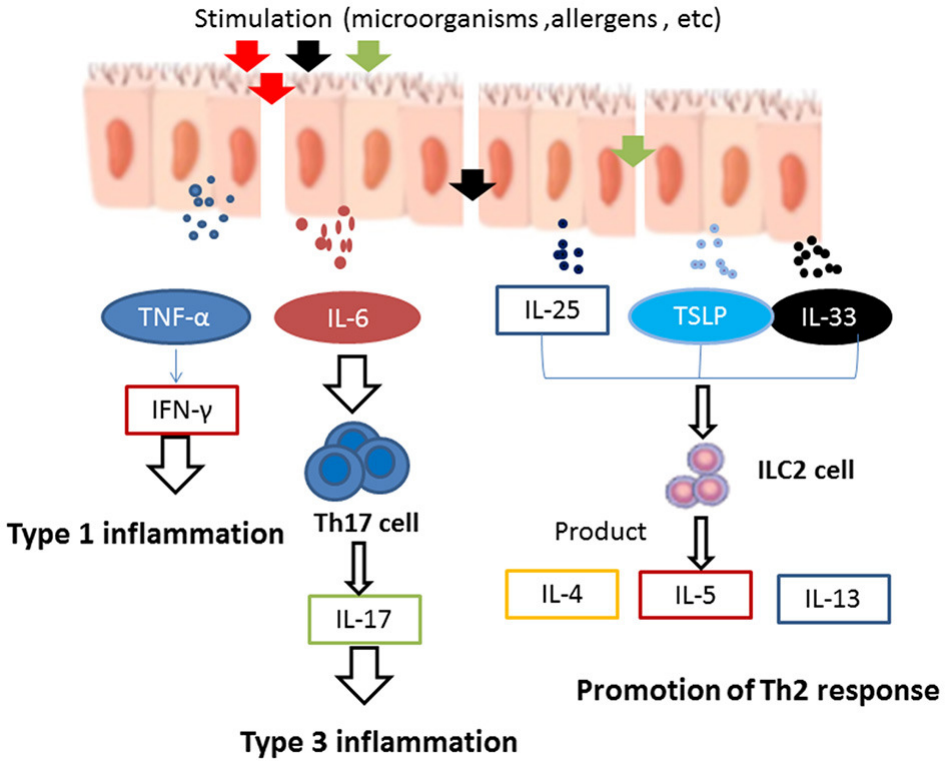
When exposed to foreign irritants, nasal epithelial cells secrete a wide variety of inflammatory cytokines, such as TNF-α, TSLP, IL-25, IL-33, and IL-6. TNF-α can promote the release of IFN-γ. It is generally believed that CRSsNP mainly involves Th1-type inflammation and high expression of IFN-γ. Epithelial-derived cytokines, such as TSLP, IL-25, and IL-33, promote the Th2 response in the development of innate lymphoid cells (ILCs) and induce the production of Th2 cytokines, such as IL-4, IL-5, and IL-13, which can further enhance type 2 inflammation. IL-6 induces the differentiation of naive T-cells into Th17 cells and produces the type 3 cytokine IL-17, which is involved in type 3 inflammation.

Interleukin-6 is a crucial regulator of inflammation, plays a role in activating B-cells, and can promote the recruitment of neutrophils (50). In addition, IL-6 and TGF-β have been found to induce juvenile T-cells to differentiate effectively into Th17 cells to produce the type 3 cytokine IL-17 (51). IL-6 is highly expressed in CRSwNP tissues (50). After epithelial damage, IL-6 can promote sinonasal epithelial cell proliferation. It has been demonstrated *in vitro* that IL-6 can facilitate repair of the nasal epithelial cells of patients with CRSwNP. However, IL-6 may also cause the epithelium to overreact to external stimuli and contribute to the formation of nasal polyps (52) (Figure 1).

#### Exosomes

Exosomes are spherical to cup-shaped vesicles, 30–150 nm in size, that contain proteins as well as genetic materials (such as miRNA, mRNA, and DNA); they can be secreted by all types of cells (53, 54). Nasal exosomes can be sampled from the nasal mucus or nasal lavage fluid (55). It has been reported that exosomes might be involved in the local pathophysiological process and immune response in CRS. Nitric oxide (NO) is well-known to play a critical role in the immune protection of airway epithelial cells. Inhaled pathogens can activate toll-like receptor 4 (TLR4) on the nasal epithelial cells, thus resulting in the doubling of basal exosome secretion rates. These exosomes carry nearly twice the level of nitric oxide synthase 2 (NOS2) found in unstimulated exosomes and can further enhance the antibacterial ability of the nasal epithelium *via* an increase in local NO content (56). Nasal mucus-derived exosomes have been found to contain various defensive proteins that can aid in strengthening the immune defensive barrier function of the sinonasal mucosa. In addition, studies have found that mucus-derived exosomes rich in P-glycoprotein (P-gp) can transfer P-gp between epithelial cells, causing type 2 helper T-cell inflammation (57). Nasal exosome proteomic analysis has revealed that exosomes display a strong association with the immune function of the nasal mucosa. A variety of immune cells, such as monocytes, NK cells, and neutrophils, can be attracted to the nasal cavity by exosomes (58). Ganglioside GD3, found on the surface of nasal mucus-derived exosomes, has the capacity to prevent T-cell receptor (TCR)-mediated T-cell activation and plays a key role in immunosuppression (59). Interestingly, a study involving proteomic analysis of the nasal mucus exosome in patients with CRSwNP has indicated that the unique bioimprints of cystatin-SN, peroxidase-5, and glycoprotein VI in the exosomes of CRSwNP nasal mucus could facilitate accurate prediction of the occurrence of this disease (60). In addition, cystatin levels in exosomes may be predictive of CRS phenotype, CRS severity, and early recurrences (61, 62). Exosomes have also been found to be associated with nasal polyp formation. A study of proteins in tissues and mucus-derived exosomes has indicated that coagulation-related tissue proteins, including fibronectin and fibrinogen gamma chains, are the most overexpressed, and fibrinolytic pathway-related proteins, including plasminase and tissue plasminogen activator, are generally downregulated in CRSwNP tissues. These observations suggest that the coagulation pathway is significantly disordered within the nasal polyp tissue: specifically, the coagulation cascade is upregulated and the fibrinolysis cascade is downregulated, thus leading to retention of plasma proteins, intense edema, excessive fibrin deposition in nasal polyps, and subsequent tissue remodeling. In addition, the expression of plasmin and coagulin in exosomes is significantly negatively correlated with their expression in the tissues, possibly due to the depletion of the cellular proteins after packaging and release into exosomes (63).

#### Other mediators

Complement expression and activity are tightly regulated to avoid immune dysregulation and host tissue damage. Both inefficient activation and overstimulation of complement have been found to be associated with increased susceptibility to infection and inflammation (64). In addition, previous studies have found that patients with CRS exhibit various complement defects (65). Patients with CRSwNP have a higher level of serum complement component 3 (C3) than patients with CRSnNP, suggesting that serum C3 may be involved in the pathogenesis of NPs. The level of serum C3 in post-operative recurrent CRSwNP patients can increase to the pre-operative level, supporting the notion that a high level of serum C3 might be considered an independent factor by which patients at high risk of recurrence can be pinpointed. Post-operative change in C3 levels might have the potential to function as a predictor of nasal polyp progression (66). The cleavage products C3a and C3b of C3 derived from the nasal epithelial cells can act to dilate the blood vessels, increase vascular permeability, promote histamine release, function as chemoattractants, activate eosinophils, regulate Th2 inflammation, and are closely related to the onset of CRSwNP (67).

Vitamin D3 (VD3) is a steroid hormone with significant antiproliferative properties (68). The dysregulation of 1α-hydroxylase and vitamin D in the sinuses in CRS has been reported previously (69). Moreover, 1α-hydroxylase can convert the inactive form of 25(OH)D3 into an active form, 1,25(OH)_2_D3. Thus, the reduction of sinonasal 1α-hydroxylase and 1,25(OH)_2_D3 in patients with CRSwNP has been found to be associated with poorer SNOT22 scores and with disease severity (70). In addition, in preclinical experiments in mice, AD3 deficiency has been found to cause changes in sinonasal immunity, resulting in increased infiltration of total inflammatory cells in the nasal cavity, including upregulation of eosinophils, neutrophils, and lymphocytes and selective exacerbation of inflammation (71). Although VD3 deficiency has been found to be associated with more severe disease, a clinical trial has demonstrated that supplementation of VD3 in patients with CRS does not significantly improve patient outcomes (72). In addition, recent studies have identified a link between complement activation and VD3. Under various stimuli, C3 and C3a might be released, which can inhibit 1α-hydroxylase expression and suppress the conversion of 25(OH)D3 to 1,25(OH)_2_D3, thus explaining the limited benefits of oral VD3 treatment (73).

### Epithelial-to-mesenchymal transition

The process by which adherent epithelial cells are converted to migratory cells invading the extracellular matrix is known collectively as EMT (74). It can occur in both physiological and pathological conditions, participating in several biological processes such as embryogenesis, wound healing, inflammation, fibrosis, and tumor metastasis (75). The phenomenon of EMT has been observed in patients with CRS and there is also evidence that it affects patients with CRSwNP. For instance, a recent study has indicated that the macrophage-derived mucin domain 4 (TIM-4) can facilitate nasal polyp formation by promoting transforming growth factor-β (TGF-β1)-mediated EMT in the nasal epithelial cells (76). It has also been discovered that TGF-β1 can induce the process of EMT *via* modulation of various microRNAs, such as microRNA-182 and microRNA-21, in CRSwNP, with attendant downregulation of the epithelial marker E-cadherin and upregulation of the mesenchymal marker vimentin and of N-cadherin (77, 78).

Interestingly, further findings have revealed that abnormal expression of EMT-related markers can be observed to a greater extent in the epithelial tissues of patients with E-CRSwNP in comparison to those obtained from patients with NE-CRSwNP, suggesting that the process of EMT might be related to the acceleration of nasal polyp formation in patients with E-CRSwNP (24). Canonical Wnt signaling has been demonstrated to be activated in nasal polyps, triggering epithelial disorders such as compromised adherens junctions and reduced ciliogenesis, which can in turn stimulate cytokine release (79). Furthermore, some studies have found that elevated expression of the various Wnt-related proteins, including nuclear β-catenin, WNT3A, and cyclin D1, in nasal polyp tissues could be accompanied by the overexpression of mesenchymal markers, thus suggesting that Wnt signaling might be involved in the pathogenesis of nasal polyp formation via stimulation of the activation of EMT (80).

### Mucosal remodeling

Remodeling of the nasal mucosa in CRS primarily takes the form of epithelial injury, basement membrane thickening (BMT), epithelial goblet cell metaplasia, mucinous gland hyperplasia, extracellular matrix (ECM) collagen deposition, and so on. Mucosal remodeling is found not only in CRSwNP but also in CRSsNP (81, 82), and E-CRS has been found to be associated with strong nasal mucosal eosinophilic infiltration (83). Evidence suggests that both eosinophilia and eosinophil activation could be closely associated with CRS remodeling and mucosal damage (84). In addition, an *in vitro* study has found that eosinophil-derived neurotoxin (EDN) degranulation from eosinophils activated by IL-5 can effectively promote the secretion of matrix metalloproteinase 9 (MMP-9) from the nasal epithelium, thereby affecting epithelial regeneration and breakdown of ECM, and ultimately leading to nasal remodeling (85). ECM proteins are also involved in the maintenance of physical scaffolding, homeostasis, and regulation of inflammation of the airway mucosa (86). Periostin, an ECM protein, is induced by IL-4 as well as IL-13 and is secreted by airway epithelial cells (87). Interestingly, studies involving nasal mucosal biopsies of CRS have shown that periosteal proteins can cause eosinophilic infiltration and mediate fibrosis, thereby participating in mucosal remodeling (88). TGF-β is a pleiotropic and multifunctional growth factor that can induce fibroblast proliferation, differentiation, and fibrotic characteristics. Moreover, it can also stimulate the production of tissue inhibitors of metalloproteinase 1 (TIMP-1), thereby preventing the enzymatic breakdown of ECM (89). It has been reported that there is a significant difference between CRSsNP and CRSwNP in terms of TGF-β levels, with higher levels detected in CRSsNP along with thicker collagen fibers in the extracellular matrix, leading to excessive tissue repair as well as the formation of fibrosis. In contrast, TGF-β is absent in CRSwNP and tissue repair is compromised, which is reflected by the presence of loose connective tissue and edema formation in the severely inflamed tissues (90).

### Autophagy

Autophagy is a lysosomal degradation pathway that is essential for cell survival, differentiation, and homeostasis. Intracellular cargo is delivered to the lysosomes and further degraded through the formation of autophagosomes (91–93). It has been established that, for protection against airborne PM2.5, smoke, and other harmful stimuli, epithelial cells may endocytose small amounts of harmful substances and trigger autophagy in airway epithelial cells, thereby increasing mucus secretion (94). However, if the induction of autophagy is insufficient to reset the cellular state to normal levels, it will further develop or even aggravate CRS (91). In the case of human neutrophil elastase (HNE)-induced CRS, studies have found that human neutrophil elastase can promote autophagy through TNF receptor-associated factor 6 (TRAF6), thus resulting in hyperexpression of MUC5AC in CRSwNP (95). Autophagy has been found to increase the secretion of MUC5AC by promoting the phosphorylation of c-Jun N-terminal kinase (JNK) and c-Jun (96). Another study of eosinophilic and non-eosinophilic nasal polyps has found that the reduction of autophagy is negatively correlated with the severity of eosinophilic inflammation and the extent of tissue remodeling (97). Other evidence suggests that autophagy in the myeloid cells, particularly macrophages, could be disrupted by the deletion of autophagy-related gene 7 (Atg7) and could further induce eosinophilic inflammation and the Th2 response, resulting in eosinophilia, epithelial hyperplasia, and mucosal thickening in E-CRS mice (98).

### Impact of therapeutic approaches on the sinonasal epithelium

It has been established above that the pathogenesis of CRS is intricate and multifactorial. For this reason, a large proportion of patients with CRS exhibit limited therapeutic benefits. As the defensive barrier of the nasal mucosa, dysfunction of the epithelium has vital significance for CRS pathophysiology. The impact of traditional and developing therapeutic approaches on the sinonasal epithelium is discussed in this section.

### Corticosteroids

Corticosteroids have well-known anti-inflammatory effects and generally suppress adaptive responses and enhance innate responses (99). The medical treatment of CRS mainly involves the application of topical, intranasal, and oral corticosteroids (100). Corticosteroids predominantly act on the glucocorticoid receptors (GCRs) distributed in sinonasal epithelial cells and exert powerful anti-inflammatory effects (101). To date, four distinct isoforms of GCR, namely, GCRα, GCRβ, GCRδ, and GCRγ, have been identified. The active isoform GCRα, after combining with corticosteroids, can bind with DNA and affect the transcription of various genes associated with inflammation, thus inhibiting the inflammatory process (102). However, variable expression of all the GCR isoforms can compromise the efficacy of corticosteroid treatment. A number of prior studies have confirmed that corticosteroid treatment can promote epithelial repair, reduce eosinophil infiltration, and affect collagen deposition, thus playing an important role in modulating mucosal remodeling (103, 104). Corticosteroids can also suppress eosinophils and Th2 cells but are relatively less effective on neutrophils and the Th17 subset (105). Intranasal corticosteroids are considered the first-line medical treatment for CRS patients after surgery. In contrast to a simple nasal spray, the use of corticosteroids delivered *via* nasal irrigation for the management of CRS is supported by a higher level of scientific evidence. For instance, an RCT in patients after sinus surgery showed that corticosteroids delivered *via* nasal irrigation can significantly improve inflammatory symptoms, such as nasal congestion and nasal discharge, and Lund-Mackay score (101). In addition, another retrospective study has demonstrated that long-term, daily intranasal corticosteroid treatment does not cause intraocular pressure, subcapsular cataracts, or other adverse effects (106). A meta-analysis has shown that, compared to placebo treatment or no treatment, the administration of a short course of oral steroids as an adjunct therapy to intranasal corticosteroids can effectively alleviate CRS symptoms, improve the microenvironment of the sinuses, and reduce the size of nasal polyps (107). The exhalation delivery system with fluticasone (EDS-FLU), a new tool for treating CRS, employs an “exhalation delivery” mechanism, exploiting balanced closure of the soft palate to deliver corticosteroids widely in order to reach key anatomical sites in the superior and posterior aspects of the nasal cavity (108). Interestingly, an RCT comparing EDS-FLU with EDS-placebo in patients with CRSwNP showed that EDS-FLU significantly improves nasal obstruction, facial pain, rhinorrhea, and hyposmia; reduces polyp size; and improves sinonasal symptoms and quality of life, while adverse events are similar to those occurring with other intranasal steroids, with the most common being epistaxis (109).

### Nasal irrigation

Nasal irrigation is regarded as an effective treatment adjunct and primarily functions to dilute the mucus, remove infective pathogens and inflammatory mediators, reduce edema of the sinonasal mucosa as well as antigen load, and improve mucociliary clearance capacity (110). The various drugs used in current nasal irrigation treatments include corticosteroids, saline, and traditional Chinese medicine (111, 112). For post-operative patients with CRS, topical steroid irrigation is especially recommended for its effectiveness in relieving various inflammatory symptoms, especially nasal congestion (106). For instance, one study has shown that isotonic saline irrigation can eliminate the antimicrobial peptides secreted by sinonasal epithelium, thus weakening the innate defensive capability of the nasal mucosa. However, low-salt-solution rinses might contribute to the secretion of various antimicrobial components (113). A meta-analysis on saline irrigation indicates that hypertonic saline, in contrast to isotonic saline, can enhance the TER levels of sinonasal epithelial cells to a remarkable degree and increase ciliary movement, thereby indicating that hypertonic saline rinses should be able to bolster the barrier function of the sinonasal mucosa (14). However, an *in vitro* study has reported that hypertonic solution rinses do substantial harm to sinus epithelial cells (114); nevertheless, detrimental effects of hypertonic solution rinses in patients have not been reported. Nasal irrigation with traditional Chinese medicine has gained widespread attention in recent years; this has unique advantages in the prevention and treatment of CRS. A number of studies have found that local nasal irrigation with traditional Chinese medicine can effectively reduce mucosal edema and promote the recovery of the structure and motor function of cilia with high levels of safety and no obvious adverse reactions (112). Honey has antibacterial and anti-inflammatory properties; thyme and honey nasal spray has been demonstrated to reduce inflammation and polyp formation as well as promoting mucosal healing in patients with CRS after surgery (115).

### Sinus surgery

Surgery is often recommended for patients experiencing failure of medical treatment. Endoscopic sinus surgery (ESS) has emerged as an optimal choice among current surgical interventions (105). A study involving face-to-face interviews with patients with CRSwNP who had undergone at least one ESS found that these patients tend to focus on the symptoms that caused their quality of life (QOL) to decline. Headaches and nasal congestion appear to impact the QoL of these patients. The most common rhinological symptom is decreased sense of smell/taste, which is a major source of distress, suggesting that clinicians should discuss CRS patients' goals and expectations for ESS with them before surgery (116). In addition, patients with CRS also suffer from poor sleep quality and reduced QOL. A meta-analysis has found that patients with CRS who undergo ESS exhibit significantly improved Pittsburgh Sleep Quality Index (PSQI) scores and QOL, although there is no significant improvement on several objective indices, including total sleep time, sleep latency, and awakenings after sleep onset (117, 118). Moreover, studies have found that the functions of the cilia and epithelial cells of patients with CRS exhibit some improvement 6 months after ESS, whereas MCC function shows some improvement 3 months after ESS (119, 120). ESS could also relieve obstruction of the nasal airway and sinus ostium, debride inflamed tissue, reestablish MCC, and provide suitable access for topical medication (105).

Failure of surgery for CRS is typically defined as the persistence or recurrence of symptoms, which often necessitates revision surgery. According to a study of 29,934 patients followed for 9.7 years, the average time between the first and second surgeries is 4.39 years, and the average time between the fourth and fifth surgeries decreases to 2.18 years (121). The average time between operations decreases as the number of revision surgeries increases. Risk factors for revision sinus surgery include phenotype, comorbidities, and surgical approach (120). For example, DeConde et al. found that polyp recurrence is common after ESS with control of polyps for up to 18 months, occurring in ~60–70% of patients with CRSwNP (122). A retrospective study of 424 adult patients with CRS receiving ESS revealed that, in an analysis by CRS subtype, revision ESS rates were 5.1% and 3.5% for CRSwNP and CRSsNP, respectively (123). Patients with CRSwNP are more likely to undergo multiple revision surgeries. Cytokine profile analysis has shown that the levels of Th17-associated mediators (IL-8, IL-17A, and IL-23), B-cell activating factor (BAFF), and Th1 cytokine (INF-γ) are significantly upregulated in the NPs of recurrence patients with CRSwNP in comparison to controls and a primary NP group (124). A meta-analysis has also shown that patients with CRSwNP who have aspirin-aggravated respiratory disease (AERD) exhibit a higher revision rate of 27.2% in comparison to various other types of patients with CRSwNP (125). In another study, Gill et al. followed 33,090 patients (7,693 with CRS with asthma (CRS-A) and 25,397 with CRS only) for 9.8 years and found that the rate of revision ESS in CRS-A was double that of patients with CRS alone; patients with CRS-A and nasal polyposis are 6 times more likely to require revision than those with CRS alone (126).

### Monoclonal biological therapies

Chronic rhinosinusitis with nasal polyps (CRSwNP) frequently remains uncontrolled despite the administration of available therapies and sinonasal surgery. Biological therapies targeting specific antagonists of pro-inflammatory mechanisms appear to be promising for personalized treatment of the inflammatory disease CRS (127). For instance, IL-5 is a potent stimulator of eosinophilic growth as well as proliferation, and the concentration of IL-5 is significantly higher in polyp tissues. A phase 3 trial of mepolizumab, an anti-IL-5 biologic, in the treatment of CRSwNP found that, after administration of mepolizumab subcutaneously at a dose of 100 mg every 4 weeks for 52 weeks (in addition to primary care), total endoscopic nasal polyp scores and nasal obstruction VAS scores significantly improved, indicating marked improvement in nasal polyp size and a reduction in polyp recurrence (128). The epithelial-derived cytokine TSLP plays a key role in inflammatory signaling and the response of congenital lymphoid cells. It can activate the Th2 response and is associated with the pathogenesis of asthma (129). Tezepelumab, a monoclonal antibody targeting TSLP, received approval in 2021 for the treatment of asthma, chronic obstructive pulmonary disease (COPD), and CRSwNP (130). In a phase 2 clinical trial, tezepelumab administered subcutaneously at 210 mg every 4 weeks for a planned 28 weeks was found to significantly reduce eosinophil counts and decrease the frequency of asthma exacerbations; it therefore represents an effective add-on treatment option for patients with CRS-A (131). Dupilumab is a monoclonal antibody that can inhibit signaling of IL-4 and IL-13, two cytokines central to Th2-mediated inflammation. In a prospective study of patients with CRSwNP who had AERD, dupilumab (at a dose of 300 mg administered subcutaneously every 2 weeks for 24 weeks, in addition to standardized treatment) was found to significantly improve scores on the SNOT-22, the asthma control test (ACT), and the Mini Asthma Quality of Life Questionnaire (AQLQ), as well as improving sinus opacification and markers of T2 inflammation. It can therefore be used as an effective therapy for these patients (132). Moreover, another RCT has demonstrated a significantly reduced polyp burden after 16 weeks of weekly subcutaneous injection of dupilumab 300 mg compared with the use of mometasone nasal spray alone (133). When epithelial cells are re-exposed to an allergen, IgE can bind to the surface receptors of both the mast cells and basophils, thus triggering degranulation of inflammatory mediators (histamine, prostate, leukotrienes, etc.) and producing various nasal symptoms (134). Omalizumab, an anti-IgE biologic agent, can produce broad therapeutic effects in patients with CRSwNP, regardless of whether they have undergone surgery, suffer from asthma, or have aspirin sensitivity (135). It can significantly improve the symptoms of nasal obstruction, runny nose, and so on in patients with CRSwNP who have an insufficient intranasal steroid response (136). The efficacy and safety of omalizumab could be extended to 1 year (137). IL-33 is involved in promoting the process of TH2 differentiation and is a key inflammatory cytokine that can mediate eosinophilic infiltration (138). Etokimab, a monoclonal antibody targeting IL-33, will be evaluated for use in patients with CRSwNP in an upcoming phase 2 controlled trial (139). These drugs offer a novel opportunity to directly target the inflammatory pathway of patients with CRS once clear biomarkers have been identified. Thus, it is possible that, once the endotypes of CRS are more thoroughly understood, personalized treatment can be further refined for the benefit of patients.

## Conclusion

Chronic rhinosinusitis is a multifactorial disease with limited treatment options. Epithelial disorder is pivotal in the etiology or pathogenesis of CRS; epithelial barrier disruption and MCC dysfunction constitute the fundamental pathogenesis of CRS. EMT, mucosal remodeling, and autophagy can affect the sinonasal epithelium and thus can also participate in the genesis of CRS. Epithelium-derived bioactive substances such as cytokines, exosomes, and complements can play an important role in the development and progression of this disease by modulating both innate and adaptive immune responses. VD3 deficiency has been found to be associated with more severe disease, but supplementation does not significantly improve patient outcomes. In terms of treatment, glucocorticoids can effectively alleviate nasal symptoms through anti-inflammatory activities, delay the onset of remodeling, and alter the course of the disease. Nasal irrigation can reduce edema by diluting the mucus and removing inflammatory mediators. Biological agents antagonize specific targets of pro-inflammatory mechanisms, thus relieving nasal symptoms and reducing polyp size. Post-operative sinus symptoms can be improved significantly in this way, and polyp burden and edema can both be relieved. In practice, however, no treatment modalities have yet been found to be successful in restoring the mucosa to its prior physiological state. Thus, novel strategies should be developed to restore the homeostasis of the nasal mucosal epithelium for treatment of CRS. Overall, considering the importance of sinonasal epithelium-mediated immunity disorders for CRS pathogenesis, vigorous efforts should be applied to the development of novel biological agents that can antagonize the actions of inflammatory bioactive components produced by the epithelium, maintain the immune balance of the nasal mucosa, alleviate local inflammation, and reduce nasal polyp formation.

## Author contributions

YH and YF were participated in drafting. YW to supplement. TZ and HL read the manuscript and gave some opinions. All authors read and approved the final manuscript.
